# Sex differences in pharmacological interventions and their effects on lifespan and healthspan outcomes: a systematic review

**DOI:** 10.3389/fragi.2023.1172789

**Published:** 2023-05-22

**Authors:** Marie Knufinke, Michael R. MacArthur, Collin Y. Ewald, Sarah J. Mitchell

**Affiliations:** Department of Health Sciences and Technology, ETH Zurich, Zurich, Switzerland

**Keywords:** sex differences, lifespan, healthspan, systematic review, pharmacological interventions, aging, mice

## Abstract

With an increasing aging population, the burden of age-related diseases magnifies. To alleviate this burden, geroprotection has been an area of intense research focus with the development of pharmacological interventions that target lifespan and/or healthspan. However, there are often sex differences, with compounds mostly tested in male animals. Given the importance of considering both sexes in preclinical research, this neglects potential benefits for the female population, as interventions tested in both sexes often show clear sexual dimorphisms in their biological responses. To further understand the prevalence of sex differences in pharmacological geroprotective intervention studies, we performed a systematic review of the literature according to the PRISMA guidelines. Seventy-two studies met our inclusion criteria and were classified into one of five subclasses: FDA-repurposed drugs, novel small molecules, probiotics, traditional Chinese medicine, and antioxidants, vitamins, or other dietary supplements. Interventions were analyzed for their effects on median and maximal lifespan and healthspan markers, including frailty, muscle function and coordination, cognitive function and learning, metabolism, and cancer. With our systematic review, we found that twenty-two out of sixty-four compounds tested were able to prolong both lifespan and healthspan measures. Focusing on the use of female and male mice, and on comparing their outcomes, we found that 40% of studies only used male mice or did not clarify the sex. Notably, of the 36% of pharmacologic interventions that did use both male and female mice, 73% of these studies showed sex-specific outcomes on healthspan and/or lifespan. These data highlight the importance of studying both sexes in the search for geroprotectors, as the biology of aging is not the same in male and female mice.

**Systematic Review Registration:** [website], identifier [registration number].

## Introduction

The world population is aging. Life expectancy has increased by 30 years over the last century (Olshansky, 2018) and in 2018, people over 65 years of age outnumbered children below 5 years for the first time (Shetty, 2012; United Nations, 2019). This demographic shift is predicted to continue, as the number of people over 65 years old is expected to triple by 2050 (Shetty, 2012). The extension of human lifespan does not always guarantee an extension of healthspan (defined as the period free from disease), as the two are not necessarily linked (Prince et al., 2015; Fischer et al., 2016; Hansen and Kennedy, 2016; Mitchell et al., 2016; Atella et al., 2019; GBD Ageing Collaborators, 2022; GBD Ageing Collborators, 2022; Statzer et al., 2022). This is demonstrated by a global increase in disease burden related to old age which goes hand in hand with the rise of the aging population (Prince et al., 2015; Atella et al., 2019; GBD Ageing Collborators, 2022; GBD Ageing Collborators, 2022). Research has focused on understanding the biological mechanisms of aging in hope of finding ways to extend lifespan and healthspan (Sinclair, 2005; Sierra, 2016; Weir et al., 2017; Olshansky, 2018). For many, extending the years lived in good health with a reduced burden of chronic diseases is a more actionable and perhaps more attractive goal than an extended lifespan (Sierra, 2016; Olshansky, 2018; Mitchell et al., 2019; Aon et al., 2020).

While lifespan was classically considered the gold standard for determining the success of geroprotectors, over the last 10 years, researchers have started to include differential measures of healthspan in their studies. The concept of frailty as a state of overall decline is increasingly utilized to assess the risk of disease and mortality in old age (Kane and Howlett, 2017; Rockwood et al., 2017; Palliyaguru et al., 2019). Tools for assessing frailty in mice (Liu et al., 2014; Whitehead et al., 2014; Hession et al., 2022), which have been reverse-translated from human scales, have become more widely utilized in recent years as markers of healthspan (Sukoff Rizzo et al., 2018; Bellantuono et al., 2020; Palliyaguru et al., 2021a) and have been shown to be modifiable by dietary and pharmacological interventions that increase lifespan (Kane et al., 2016; Palliyaguru et al., 2019). Beyond the mouse frailty index, other important assays to assess health broadly span the domains of muscle function and coordination, cognitive function and memory, metabolic function, and tumor incidence (Ackert-Bicknell et al., 2015; Bellantuono et al., 2020). While there is no established stringent set of measures agreed upon by the entire community to fully define healthspan in mice, a number of important publications have established at least a panel of markers with demonstrated utility in the assessment of healthspan (Richardson et al., 2016; Bellantuono et al., 2020).

Interventions to increase lifespan and healthspan comprise behavioral, dietary, and pharmacological approaches (Longo et al., 2015), and are commonly referred to as geroprotectors (Moskalev et al., 2016). Potential geroprotectors are defined as interventions which may extend lifespan and/or healthspan by targeting one or more of the hallmarks of aging (Moskalev et al., 2016; Janssens and Houtkooper, 2020; López-Otín et al., 2023). Examples of successful geroprotectors include rapamycin and metformin (Martin-Montalvo et al., 2013; Bitto et al., 2016; Glossmann and Lutz, 2019; Selvarani et al., 2020; Moskalev et al., 2022). The development of geroprotectors is based on the “Geroscience hypothesis” (Sierra and Kohanski, 2017), in which aging plays a central role in many, if not all, chronic diseases. Interventions that retard aging should simultaneously delay the onset of many diseases according to this hypothesis. This foundational framework has proposed a roadmap for how geroprotectors should impact aging. A number of recent reviews have eloquently described the role of dietary interventions as potential geroprotectors (Brandhorst and Longo, 2019; Green et al., 2022; Longo and Anderson, 2022; Mitchell and Mitchell, 2022), so they will not be included here.

It is well-known that there are sexual dimorphisms in the aging process, including in healthspan, muscle mass maintenance and physical performance, sex-hormones, age-related diseases and lifespan (Austad and Fischer, 2016; Le Couteur et al., 2018; Sampathkumar et al., 2019; Decaroli et al., 2021; Bronikowski et al., 2022; Viecelli and Ewald, 2022; Della Peruta et al., 2023). It is noteworthy that men and women have different susceptibility to various age-related diseases, such as women being more likely to develop osteoporosis, and men being more prone to cardiovascular diseases (Crimmins et al., 2019). This is partly influenced by sex-specific alterations in sex hormones with age, including a decrease in estrogen levels during menopause for women and a decline in testosterone with age for men (Guarner-Lans et al., 2011; Horstman et al., 2012; Decaroli et al., 2021). Further, women and females of other species tend to have significantly longer lifespans, but experience higher levels of frailty at a given age when assessed clinically (Le Couteur et al., 2018; Gordon and Hubbard, 2019; Kane and Howlett, 2021). In mice, sex-related differences can be seen in physical performance, which was shown to be lower in aging males (Tran et al., 2021), while anxiety-like behaviors were increased in aging males (Kobayashi et al., 2021). Even on the tissue and molecular level, there are vast sex-specific differences in mice’s gene expression signatures associated with longevity (Vitiello et al., 2021). Further, sexual dimorphisms can be observed in geroprotective interventions aiming to increase lifespan or healthspan (Sampathkumar et al., 2019). Both dietary and pharmacological interventions, such as rapamycin and calorie restriction, have been shown to have sexually dimorphic effects when tested in mice (Anisimov et al., 2010; Harrison et al., 2014; Miller et al., 2014; Mitchell et al., 2016; Bielas et al., 2018; Cabo and Mattson, 2019; Sampathkumar et al., 2019; Berry et al., 2020; Henderson et al., 2021). These findings suggest underlying biological differences in the mechanisms of aging between the sexes and highlight the importance of considering sex as a biological variable. Despite a 2016 NIH mandate requiring both sexes to be used in preclinical research (NOT-OD-15-102, 2015: Consideration of Sex as a Biological Variable in NIH-funded Research,” 2015), many fields, including the aging field, still face challenges to the inclusion of both sexes in their studies (Plevkova et al., 2020; Shansky and Murphy, 2021; Carmody et al., 2022; Merone et al., 2022). To comprehensively compile the current literature and provide a summary of findings, we performed a systematic review of original research publications from 1970 to 2022 and reviewed what is known about sexual dimorphisms in the lifespan and healthspan outcomes of mice undergoing some form of pharmacological intervention. Our findings are presented herein.

## Methods

**FIGURE 1:**
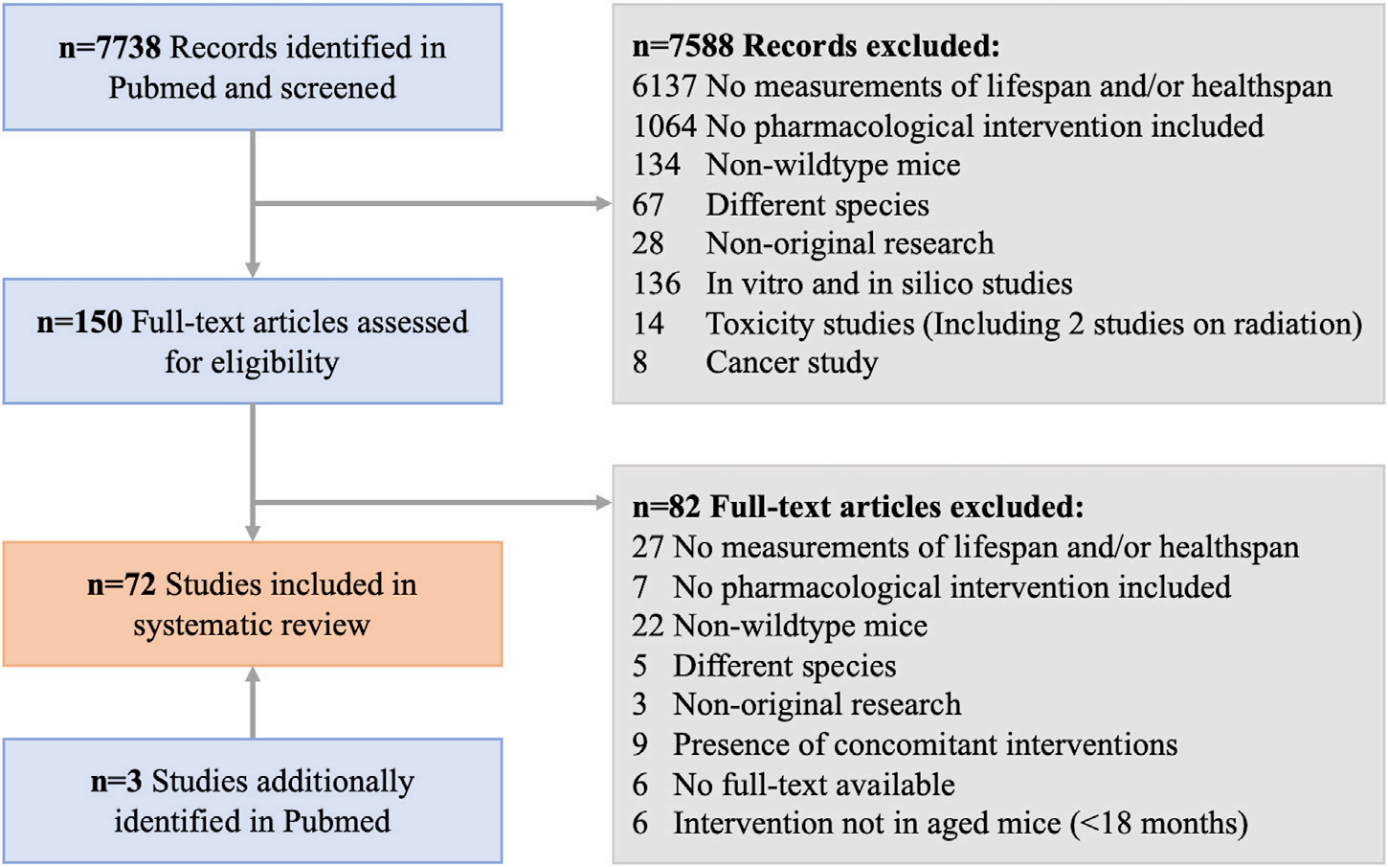
Workflow for the systematic review from identified records to all included studies in this manuscript.

A systematic review of the literature was conducted according to the PRISMA guidelines (Page et al., 2021) to identify publications reporting on pharmacological interventions in mice and their effects on lifespan and/or healthspan, in a sexually dimorphic manner. PubMed (RRID:SCR_004846) was utilized as the search tool and database to screen the title, abstract, and keywords of all articles (excluding reviews) using the search terms with Boolean operators as outlined in Table 1. The search period was limited to all published within the period of 1970 to 1. January 2022. All identified records were exported to excel, where the authors (M.K. and S.J.M) screened them for the eligibility criteria and removed duplicate records, irrelevant titles/abstracts, as well as non-original research (re-analysis of previously published data, commentaries, etc.). To ensure all relevant research was included, an additional manual review of the literature was performed via PubMed, which produced four further studies. Of the remaining potential records, the full-text articles were screened against the exclusion criteria (Table 1) and the resulting 72 eligible articles were used as the basis for this systematic review. Figure 1 shows the workflow for the literature search and study selection, including the identification of the final 150 full-text articles, of which 72 were included in the final analysis. From the final articles, we extracted all relevant information for this review. This included details of the study design as well as the study outcomes for lifespan and healthspan parameters. For the study design, the compound used in the intervention, its dose and method of administration, the age at onset of the intervention and its duration, and the mouse strain used were recorded. The lifespan measures were separated into measures of median lifespan and maximal lifespan due to inherent differences in how authors report these findings. Due to the diversity of the healthspan measurements in mice, we defined healthspan parameters according to published recommendations (Richardson et al., 2016; Bellantuono et al., 2020) to include frailty, muscle function and coordination, cognitive function and memory, metabolic function, and cancer incidence. The limitation of these assays is the missing consensus of what measure(s) reliably demonstrate improvements in healthspan. For all outcomes it was reported whether there was a significant improvement (↑) or worsening (↓) of the parameter during the study in the intervention group relative to the control group stratified by sex. We also reported whether there was a sexual dimorphism (defined as opposing directionalities of the effect, i.e., improved in males, worsened in females) in the measured outcome. If the outcome was not reported in the respective study this was denoted with a “n.m.” (not measured). For median and maximal lifespan, *p*-values were added if reported by the authors. All the information was then structured according to the drug class of the compound used, covering repurposed FDA drugs, novel small molecules, probiotics, traditional Chinese medicine, and supplements, including vitamins and antioxidants.

**TABLE 1:** Search strategy and eligibility criteria.

Search strategy	Eligibility criteria	
	Exclusion criteria	Inclusion criteria
Healthspan OR (health AND span) OR health span	No measurements of lifespan and/or healthspan	Lifespan and/or healthspan measured
AND longevity OR longevities OR lifespan OR lifespans OR mortality OR survival OR survivability OR survivable OR survivals OR survive OR survived OR survives OR surviving	No pharmacological intervention included	Only pharmacological studies included
AND male OR males OR (male AND female) OR female OR females	Non-wildtype mice	Only wildtype animals
AND English	Different species	In mice and/or rats
NOT review OR review literature as topic	Non-original research	Original research
NOT human OR humans	*In-vitro* and *in silico* studies	No *in-vitro* or *in silico* analysis
AND mice OR rats	Toxicity studies	Male and/or female animals
NOT in vitro NOT cell NOT clinical	Cancer study	Text in English
	No full-text available	Full text available via PubMed
	Presence of concomitant interventions	
	Intervention not in aged mice (<18 months)	

## Results

**FIGURE 2:**
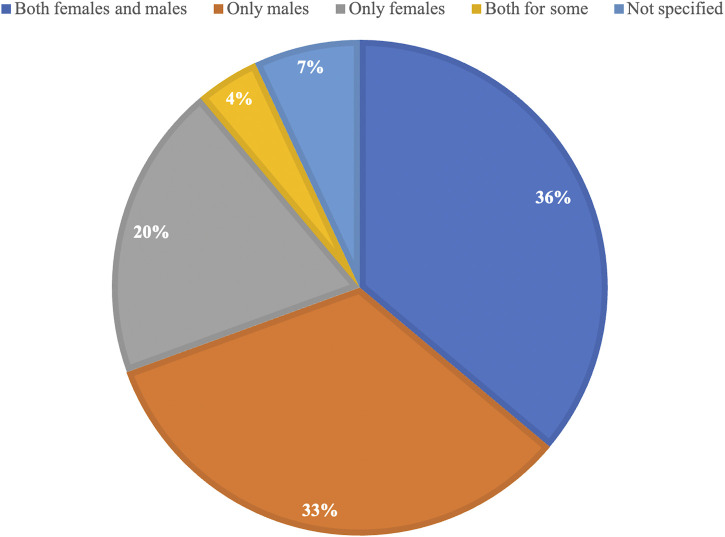
Proportional representation of the use of female and male mice in all studies included in this manuscript.

The final 72 eligible studies (Figure 1) that were included in this review and published between 1970 and 2022 reveal that 36% (26/72) included both female and male mice in their research, while 33% (24/72) used only male mice and 20% (14/72) used only female mice (Figure 2). Out of the 26 studies including both sexes, a large part showed sex-specific results (19/26). Next to measurements of lifespan, a wide variety of healthspan metrics started to be included in studies from the year 2000 onwards, with a continuous increase in their implementation over time. All but one study conducted since 2020 included some sort of healthspan parameter. Of all compounds tested, 22 out of 64 were able to show positive effects on both lifespan and healthspan measures.

### Repurposed FDA drugs

Repurposing FDA drugs for use in age-related diseases has been a popular strategy for identifying new geroprotectors (Table 2). One of the attractive benefits of this strategy is the wealth of pharmacology, safety, and efficacy data already available for these compounds. This means investigators can direct resources to validating the compound in the appropriate model, rather than re-hashing safety data that already exists. Many proposed geroprotectors have been tested in the NIA Interventions Testing Program (ITP) in the United States (Miller et al., 2007). This program solicits investigator-proposed compounds and tests their lifespan potential in genetically heterogeneous UM-HET3 mice. Started in the early 2000s, this program to date has tested more than 60 different compounds.

**TABLE 2:** Study details and results for interventions with repurposed FDA drugs.

Study	Compound and dose	Start-age and duration	Strain	Outcome lifespan		Outcome healthspan				
				Median lifespan	Max lifespan	Frailty	Muscle function and coordination	Cognitive function and learning	Metabolism	Cancer
Zhu et al. (2020)	Metformin, 100 mg/kg/day (water)	20 months, until end of life	No info	↓ f (*p* < 0.05)	n.m	n.m	n.m	n.m	n.m	n.m
Hiramoto et al. (2020)	Tranexamic acid, 12 mg/kg 3 times a week (water)	2 months, for 24 months	ICR-CD1	n.m	n.m	n.m	↑ m	↑ m	n.m	n.m
Sciorati et al. (2020)	Etanercept, 1 mg/kg/week (injection)	16 months, for 12 months	C57BL/6	n.m	↑ f (*p* = 0.028)	n.m	↑ f	n.m	n.m	n.m
Miller et al. (2020)	Canagliflozin, 30 mg/kg/day (food)	7 months, until end of life	UM-HET3	↑ 14% m (*p* < 0.001)	↑ 9% m (*p* < 0.001)	n.m	n.m	n.m	↑ f and m	= f and m
				= f	= f					
Strong et al. (2020)	Rapamycin, 42 mg/kg/day (food)	20 months, until end of life	UM-HET3	↑ 11% m (*p* < 0.001)	↑ 9% m (*p* = 0.04)	n.m	n.m	n.m	n.m	n.m
				↑ 15% f (*p* < 0.0001)	↑ 12% f (*p* < 0.0001)					
		20 months, 1-month cycles until end of life		↑ 9% m (*p* = 0.002)	↑ 9% m (*p* = 0.001)					
				↑ 8% f (*p* < 0.0001)	↑ 10% f (*p* < 0.001)					
		20 months, for 3 months		↑ 11% m (*p* < 0.024)	= m (*p* = 0.08)					
				= f (*p* = 0.15)	= f (*p* = 0.12)					
Palliyaguru et al. (2020)	Metformin, 500 mg/kg*bw/day (food) + HFD	14 months, until end of life	C57BL/6 J	= m	= m	n.m	↑ m	n.m	n.m	n.m
	Metformin 500 mg/kg*bw/day + SRT1720 100 mg/kg*bw/day (food) + HFD			↓ m (*p* < 0.0001)	↓ 35% m (*p* < 0.0001)		↑ m			
Harrison et al. (2021)	Candesartan cilexetil, 30 ppm	8 months, until end of life	UM-HET3	= f and m	= f and m	n.m	n.m	n.m	n.m	n.m
Hiramoto et al. (2019)	Tranexamic acid, 12 mg/kg 3 times a week (water)	2 months, until end of life	Hairles mouse (Hos:HR-1)	n.m	↑ m (*p* < 0.01)	n.m	n.m	n.m	n.m	↑ m
Smith et al. (2019)	Acarbose, 1,000 ppm (food)	8 months, until end of life	UM-HET3	↑ 5% f (*p* = 0.003)	n.m	n.m	n.m	n.m	n.m	n.m
				↑ 17% m (*p* < 0.001)						
Bielas et al. (2018)	Rapamycin, 14 ppm (food)	9 months, for 13 months (careful, control under 40% dietary restriction)	UM-HET3	n.m	n.m	n.m	= f and m	n.m	n.m	n.m
	Rapamycin, 42 ppm (food)						= f and m			
Thangthaeng et al. (2017)	Metformin, 219–297 mg/kg/day (water)	22 months, for 3 months	C57BL/6 J	n.m	n.m	n.m. (with met visual acuity decreased)	= m (with met took longer to initiate walking)	= m (with met spatial memory worse)	= m	n.m
Bitto et al. (2016)	Rapamycin, 8 mg/kg/day (intraperitoneal)	20–21 months, for 3 months	C57BL/6JNia	↑ 14% m (*p* = ?)	n.m	n.m	n.m	n.m	n.m	↓ f
				= f						
	Rapamycin, 126 ppm (food)			↑ 14% m (*p* = ?)	n.m	n.m	↑ f and m	n.m	n.m	n.m
				↑ 9% f (*p* = ?)						
Fischer et al. (2015)	Rapamycin, 14 ppm (food)	4 months, until end of life	C57BL/6 J	n.m	n.m	n.m	↑ f (grip strength, ↓ m (rotarod)	n.m	n.m	n.m
							= m and f (activity, stride length)			
Miller et al. (2014)	Rapamycin, 4.7 ppm (food)	9 months, until end of life	UM-HET3	↑ 16% f (*p* < 0.0001) = m (3%, *p* = 0.19)	↑ 5% f (*p* < 0.0001) = m (6%, *p* = 0.23)	n.m	n.m	n.m	n.m	n.m
	Rapamycin, 14 ppm (food)			↑ 21% f (*p* < 0.0001) ↑ 13% m (*p* = 0.0015)	↑ 11% f (*p* < 0.0001) ↑ 8% m (*p* = 0.003)					
	Rapamycin, 42 ppm (food)			↑ 26% f (*p* < 0.0001) ↑ 23% m (*p* < 0.0001)	↑ 11% f (*p* < 0.0001) ↑ 8% m (*p* = 0.004)					
Harrison et al. (2014)	Acarbose, 1,000 ppm (food)	4 months, until end of life	UM-HET3	↑ 5% f (*p* = 0.01)	↑ 9% f (*p* = 0.001)	n.m	↑ f	n.m	= f	n.m
				↑ 22% m (*p* < 0.0001)	↑ 11% m (*p* < 0.001)		= m (activity)		↑ m (insulin reduced, but higher fasting glucose)	
Zhang et al. (2014)	Rapamycin, 14 ppm (food)	19 months, until end of life	C57BL/6Nia	n.m	↑ f (*p* = 0.047)	n.m	↑ f and m (f more active, ↑ gait and rotarod, not grip)	n.m	n.m	↑ f (fewer neoplastic lesions and adenomas, careful interpretation) = m
					= m (*p* = 0.275)					
Flynn et al. (2013)	Rapamycin, 14 ppm (food)	24 months, for 3 months	C57BL/6 J	n.m	n.m	n.m	↑ f (activity)	n.m	= f (after 3 months, initial increase in glucose)	n.m
Neff et al. (2013)	Rapamycin, 14 ppm (food)	4 months, for 12 months	C57BL/6 J Rj	n.m	n.m	n.m	↑ m (exploration OF, no effect grip strength)	↑ m (not in object rec., but in maze and far cond., for all)	n.m	↑ m (less cancer)
		13 months, for 12 months		n.m	n.m	n.m	= m	↑ m	n.m	= m (careful)
		20–22 months, for 12 months		n.m	n.m	n.m	= m	↑ m	n.m	= m (careful)
Martin-Montalvo et al. (2013)	Metformin, 100 ppm (food)	12 months, until end of life	C57BL/6	↑ 5.83% m (*p* = 0.02, mean ls)	n.m	↑ m (cataracts)	↑ m	n.m	↑ m (glucose)	= m
	Metformin, 1,000 ppm (food)			↓ 14.4% m (*p* < 0.001, mean ls)	n.m	n.m	n.m	n.m	n.m	n.m
	Metformin, 100 ppm (food)		B6C3F1	= 4.15% m (*p* = 0.064, mean ls)	n.m	n.m	↑ m	n.m	n.m	= m
Wilkinson et al. (2012)	Rapamycin, 4.7 ppm (food)	9 months, for 13 months	UM-HET3	n.m	n.m	↓ f and m (only cataracts)	= f and m (only spont. activity)	n.m	n.m	↑ f and m (only adrenal, not others)
	Rapamycin, 14 ppm (food)			n.m	n.m	↓ f and m (only cataracts)	= f	n.m	n.m	↑ f and m (only adrenal, not others)
							↑ m (only spont. activity)			
	Rapamycin, 42 ppm (food)			n.m	n.m	↓ f and m (only cataracts)	↑ f	n.m	n.m	↑ f and m (only adrenal, not others)
							= m (only spont. activity)			
Majumder et al. (2012)	Rapamycin, 14 mg/kg food	2 months, for 16 months	C57BL6/129svj	n.m	n.m	n.m	n.m	↑ (?)	n.m	n.m
		15 months, for 3 months		n.m	n.m	n.m	n.m	= (?)	n.m	n.m
Smith et al. (2011)	Sibutramine, 1.25 or 5 or 20 mg/kg/day (food)	1 month, until end of life	CD-1	= f and m	n.m	n.m	n.m	n.m	n.m	n.m
Miller et al. (2011)	Rapamycin, 14 ppm (food)	9 months, until end of life	UM-HET3	↑ 18% f (*p* < 0.0001) ↑ 10% m (*p* < 0.0001)	↑ 13% f (*p* < 0.01)	n.m	↑ m (activity)	n.m	n.m	= f and m
					↑ 16% m (*p* < 0.01)		= f			
	Simvastatin, 12 or 120 ppm (food)	10 months, until end of life		= f andm	= f andm	n.m	= f andm	n.m	n.m	n.m
Anisimov et al. (2010)	Metformin, 100 ppm (water)	3 months, until end of life	129/sv	↑ 7.8% f (*p* < 0.05, median ls)	= f and m	n.m	n.m	n.m	= m (glucose, cholesterol, trigly, insulin)	↑ f (less malignant tumors)
				↑ 4.4% f (*p* < 0.05, mean ls)						= m
				↓ 13.4% m (*p* < 0.05, mean ls)						
Harrison et al. (2009)	Rapamycin, unknown dose	21 months, until end of life	UM-HET3	n.m	↑ 14% f (*p* < 0.0001) ↑ 9% m (*p* < 0.0001)	n.m	n.m	n.m	n.m	= f and m
Strong et al. (2008)	Aspirin, 21 ppm (food)	4 months, until end of life	UM-HET3	= f	= f and m	n.m	n.m	n.m	n.m	n.m
				↑ m (*p* = 0.01)						
Anisimov et al. (2008)	Metformin, 100 ppm (water)	3 months, until end of life	SHR	↑ 37.9% f (*p* < 0.01, mean ls)	↑ 10.3% f (*p* = ?)	n.m	n.m	n.m	= f	= f
				↑ 91.9% f (*p* = ?, median ls)						
Popovich et al. (2003)	Deltaran (Ibuprofen), 2.5 mg (5x per months, injection)	3 months, until end of life	SHR	= f	↑ f (*p* < 0.01, last 10%)	n.m	n.m	n.m	n.m	↑ f
Forbes (1975)	Prednisolone sodium phosphate 15–16 mg/day (water)	8 months, until end of life	DBA/2 J	= f (mean ls)	n.m	n.m	n.m	n.m	n.m	n.m
Cotzias et al. (1974)	L-Dopa	1 month, for 18 months	Swiss albino	↑ m (*p* < 0.001, only measured at 19 m)	n.m	↓ m (corneal opacity)	= m	n.m	n.m	n.m
	5,000 mg/kg*bw/day									

Notes: f, female; m, male; n.m., outcome was not measured (?), the sex was not specified; The arrows denote a significant improvement (↑) or worsening (↓) of the respective outcome in the intervention group relative to the control group, while a (=) denotes no difference to control.

One of the most promising compounds that came out of the ITP is rapamycin. Rapamycin has potent antitumor and immunosuppressive activity and was originally discovered in soil samples from the Easter Island. In mice, rapamycin has been tested at various doses, as well as at different ages of onset. Most data are consistent with the notion that rapamycin extends lifespan in both males and females, with stronger effects shown in females (Harrison et al., 2009; Miller et al., 2011; Miller et al., 2014; Zhang et al., 2014; Strong et al., 2020). Healthspan data supports the concept that age-related deficits are mitigated with rapamycin treatment (Flynn et al., 2013; Neff et al., 2013; Zhang et al., 2014), although specific tests may or may not show sexually dimorphic results (Table 2). Recent work has demonstrated that rapamycin treatment in the first 45 days of life is sufficient to improve healthspan, reduce frailty and extend median lifespan, at least in males (Shindyapina et al., 2022). Moreover, rapamycin treatment for 3 months during middle age (20–21 months) increased median lifespan by 14% for males and 9% for females (Bitto et al., 2016). This data highlights the importance of considering the age of onset of these therapeutics and that lifelong treatment may not be necessary. Other drugs tested in the ITP include aspirin, canagliflozin, candesartan, metformin, sibutramine, simvastatin, and acarbose (Strong et al., 2008; Miller et al., 2011; Smith et al., 2011; Harrison et al., 2014; Smith et al., 2019; Miller et al., 2020; Harrison et al., 2021). Canagliflozin, a diabetes drug, showed sexually dimorphic effects on lifespan, with an increase in both median and maximal lifespan of 14% and 9%, respectively, only in males (Miller et al., 2020). In a parallel study, canagliflozin was found to retard age-related lesions in males only, suggesting that the lifespan extension in the treated males is likely a reflection of delay in lethal neoplasms (Snyder et al., 2022). Interestingly, another diabetes drug, metformin, did not show lifespan extension in genetically heterogeneous males but did have small but significant effects on median lifespan in C57BL/6 J males, and a trend towards an effect in B6C3F1 male mice (Martin-Montalvo et al., 2013). Metformin at 0.1% improved markers of health in these mice, however, it must be noted that 1% metformin caused significant kidney damage and significantly reduced lifespan by 14% (Martin-Montalvo et al., 2013). Others have also tested metformin and shown that 1% metformin improved median and maximal lifespan in female SHR mice by 91.9% and 10.3%, respectively (Anisimov et al., 2008). When tested in 129/sv mice, the same concentration improved median lifespan in females by 7.8% but reduced it by 13.4% in males. In a recent study in female mice of an unknown strain, 1% metformin reduced their median lifespan (Zhu et al., 2020). Taken together, there are clear sexual dimorphic effects of metformin in different mouse strains on lifespan, with a lack of a clear directionality effect across strains. Several studies were able to show positive healthspan effects of metformin doses ranging from 1% to 5% in C57BL/6 J mice (Martin-Montalvo et al., 2013; Palliyaguru et al., 2020), illustrating the uncoupling of lifespan and healthspan outcomes. A third diabetes drug, acarbose, showed promising effects on lifespan in both female and male genetically heterogenous mice, with larger effects in males (Harrison et al., 2014; Smith et al., 2019). Healthspan was not tested in these studies. Aspirin, a classic anti-inflammatory drug, extended median lifespan in male, but not in female UM-HET3 mice (Strong et al., 2008). No effects on lifespan or healthspan were shown by the drugs Candesartan (Harrison et al., 2021), an antihypertensive drug, Sibutramine (Smith et al., 2011), an appetite suppressant, or Simvastatin (Miller et al., 2011), a statin reducing cholesterol. As these compounds were tested as part of the ITP, healthspan measures were not included in these studies.

Drugs that were tested outside of the ITP include tranexamic acid, Deltaran, Etanercept, L-Dopa, and Prednisolone. Deltaran, Etanercept, and Prednisolone are all anti-inflammatory drugs that were tested in female mice, from which the first two had positive effects on lifespan and healthspan (Popovich et al., 2003; Sciorati et al., 2020). Prednisolone showed no effects on lifespan (Forbes, 1975). L-Dopa, a precursor to the neurotransmitters dopamine, noradrenaline, and adrenaline, showed positive effects on male lifespan but had no impact on healthspan (Cotzias et al., 1974). Tranexamic acid, an antifibrinolytic, positively impacted male lifespan and healthspan parameters (Hiramoto et al., 2020; 2019).

Overall, using repurposed FDA drugs as geroprotectors is a promising strategy. Still, more research is needed to determine the optimal doses, ages of onset, and specific indications for these drugs, as well as the effectiveness in both sexes.

### Novel small molecules

Beyond repurposing already approved drugs, a common approach in drug development is developing novel small molecules, which allows for a more target-specific approach. Examples of pathways that novel small molecule may target in the aging field, include oxidative stress, inflammation, AMPK, or senescence (Table 3). These are some of the processes implicated as hallmarks of aging (López-Otín et al., 2023).

**TABLE 3:** Study details and results for interventions with novel small molecules.

Study	Compound and dose	Start-age and duration	Strain	Outcome lifespan		Outcome healthspan				
				Median lifespan	Max lifespan	Frailty	Muscle function and coordination	Cognitive function and learning	Metabolism	Cancer
Dorigatti et al. (2021)	Beta-guadinidinopropionic acid, 300 ppm ad libitum (food)	18–19 months, for 17–22 (m)/25–26 (f) weeks	UM-HET3	n.m	n.m	n.m	↑ f and m (only gait, not muscle strength)	n.m	= f and m	n.m
Palliyaguru et al. (2020)	SRT1720, 100 mg/kg*bw/day (food)	14 months, until end of life	C57BL/6 J	↑ m (*p* < 0.0001)	= m	n.m	= m	n.m	n.m	n.m
Harrison et al. (2021)	17-α-estradiol, 14ppm	16 months, until end of life	UM-HET3	↑ 19% m (*p* < 0.0001)	↑ 7% m (*p* < 0.004)	n.m	n.m	n.m	n.m	n.m
		20 months, until end of life		↑ 11% m (*p* < 0.007)	= m (*p* = 0.17)					
	Geranylgeranyl-acetone, 600 ppm	9 months, until end of life		= f and m	= f and m					
	MIF098, 240 ppm	8 months, until end of life		= f and m	= f and m					
Sun et al. (2019)	Dimethylamino-micheliolide, 10 mg/kg/EOD (orally)	12 months, for 15 months	C57BL/6	= m	n.m	n.m	↑ m (only treadmill, not rotarod)	= m	↑ m	n.m
	Dimethylamino-micheliolide, 25 mg/kg/EOD (orally)			= m	n.m	n.m	= m	↑ m	↑ m	n.m
	Dimethylamino-micheliolide, 50 mg/kg/EOD (orally)			= m	n.m	n.m	↑ m (only open field tot distance)	= m	= m	n.m
Krut’ko et al. (2016)	Alpha-fetoprotein, 10 mg/kg*bw/day (intraperitoneal)	18 months, for 2 weeks	BALB/c	n.m	n.m	↑ f (coat condition and hair loss)	↑ f (but statistics not very good)	n.m	n.m	n.m
Harrison et al. (2014)	17-α-estradiol, 4.8 ppm (food)	10 months, until end of life	UM-HET3	= f (*p* = 0.8)	= f (*p* = 0.9)	n.m	n.m	n.m	n.m	n.m
				↑ 12% m (*p* = 0.0012)	= m (*p* = 0.13)					
Mitchell et al. (2014)	SRT1720, 100 mg/kg*bw/day (food)	6 months, until end of life	C57BL/6 J	= m (trend *p* = 0.096)	= m	↑ m (less cataracts)	↑ m (improved rotarod 13 and 18 months)	n.m	↑ m (lower glucose)	= m
				↑ 8.8% m (*p* = 0.04, mean ls)						
Quick et al. (2008)	Carboxy-fullerene SOD mimetic, 10 mg/kg/day (water)	12 months, until end of life	C57BL/6	↑ 11% f and m (*p* = 0.004, mean ls, analyzed together)	↑ f and m	n.m	n.m	↑ f and m	n.m	n.m
Strong et al. (2008)	Nitroflurbiprofen, 200 ppm (food)	4 months, until end of life	UM-HET3	= f and m	= f and m	n.m	n.m	n.m	n.m	n.m
	4-OH-PBN, 350 ppm (food)	4 months, until end of life		= f and m	= f and m	n.m	n.m	n.m	n.m	n.m

Notes: f, female; m, male; n.m., outcome was not measured (?), the sex was not specified; The arrows denote a significant improvement (↑) or worsening (↓) of the respective outcome in the intervention group relative to the control group, while a (=) denotes no difference to control. EOD, every other day; SOD, superoxide dismutase; 4-OH-PBN, 4-OH-a-phenyl-N-tert-butyl nitrone.

Promising results have been shown with a carboxy-fullerene superoxide dismutase (SOD) mimetic and 17-α-estradiol. The SOD mimetic with its antioxidant properties was able to extend female and male lifespans by 11% and improved the mice’s cognition and learning (Quick et al., 2008). In the ITP, 17-α-estradiol, a synthetic form of the hormone estradiol with proposed neuroprotective properties, has been found to extend lifespan in male mice in repeated studies but not in females (Harrison et al., 2014; Harrison et al., 2021). Lifespan effects have ranged from a median lifespan increase in male mice of 12% (Harrison et al., 2014) up to 19% (Harrison et al., 2021). While healthspan was not measured in these two studies, independent studies have shown that healthspan benefits are seen in both male rats and mice with 17-a-estradiol (Mann et al., 2020), highlighting the importance of cross-species validation of potential geroprotectors.

Two small molecules, SRT1720 and SRT2104, which were developed as specific sirtuin 1 (SIRT1) activators, have shown benefits in both healthspan and lifespan measures in both a high-fat diet (HFD) background, as well as a standard diet background. Mitchell et al. found that SRT1720 improved several measures of healthspan in male mice as well as mean lifespan, but only a trend towards increasing median lifespan (Mitchell et al., 2014). More striking effects were seen when HFD-fed mice were treated with SRT1720 (Minor et al., 2011). Although in a second study of SRT1720 on an HFD, bodyweight and rotarod performance were not significantly different from the control group (Palliyaguru et al., 2020). A significant limitation of this work was that these compounds were not tested in female mice, thereby limiting the generalizability of these results.

Age-related changes in the immune system, often referred to as “inflamm-aging” (Franceschi et al., 2018), contribute to the pathogenesis of many age-related diseases. Alpha-fetoprotein, an immunoregulator, improved muscle function and coordination in female mice (Krut’ko et al., 2016). The anti-inflammatory molecules MIF098 (Harrison et al., 2021), dimethylaminomicheliolide (Sun et al., 2019), and nitroflurbiprofen (Strong et al., 2008), as well as the antioxidant 4-OH-PBN (Strong et al., 2008) and the insulin sensitivity promoting geranylgeranyl-acetone (Harrison et al., 2021) showed no effect on lifespan when tested in male and female UM-HET3 mice. Beta-guadinidinopropionic acid, an AMPK activator (Dorigatti et al., 2021), showed improvements in muscle function and coordination independent of sex when measured with gait and rotarod performance, but not with grip strength or exercise tolerance tests, while lifespan was not measured.

### Probiotics

We only identified one probiotic, *Akkermansia muciniphila*, that has been tested as a potential geroprotector and fulfilled the criteria to be included in this review. Two studies, displayed in Table 4, found that *Akkermansia muciniphila* had no effect on lifespan, but did show minor improvements in healthspan measures such as frailty, muscle function, and cognitive function in female mice (Cerro et al., 2021; Shin et al., 2021). Further research is needed to fully understand the potential of *A. muciniphila* and other probiotics as potential geroprotectors including determining the optimal dosage and administration for use in humans.

**TABLE 4:** Study details and results for interventions with probiotics.

Study	Compound and dose	Start-age and duration	Strain	Outcome lifespan		Outcome healthspan				
				Median lifespan	Max lifespan	Frailty	Muscle function and coordination	Cognitive function and learning	Metabolism	Cancer
Shin et al. (2021)	*Akkermansia muciniphila*, 4.9 × 10^8^ CFU/150 mL/day (orally)	24–25 months	C57BL/6 J	= (?)	= (?)	↑ (?)	↑ (?)	↑ (?)	n.m	n.m
Cerro et al. (2021)	*Akkermansia muciniphila*, 2 × 10^8^ CFU/100 mL/day (orally)	18 months, for 1 month	ICR-CD1	n.m	= f	n.m	= f	↑ f	n.m	n.m

Notes: f, female; m, male; n.m., outcome was not measured (?), the sex was not specified; The arrows denote a significant improvement (↑) or worsening (↓) of the respective outcome in the intervention group relative to the control group, while a (=) denotes no difference to control. CFU, stands for “Colony Forming Unit”.

### Traditional Chinese medicine

Traditional Chinese medicine (TCM) is another field where researchers have tested compounds for their effects on longevity and healthspan (Zhao and Luo, 2017) (Table 5). TCM has a long history, and many herbs and their components are being studied now in a variety of diseases where they show beneficial effects, including aging (Chen et al., 2019; Bi et al., 2022; Xue et al., 2022). The flavanol Icariin, an ingredient of the herb *Epimedium*, improved the median lifespan in male mice by 8%, accompanied by improved muscle function and coordination. These beneficial effects could be attributed to its purported anti-inflammatory and anti-oxidant properties (Zhang et al., 2015; Bi et al., 2022). A late-onset treatment (22–23 months of age) with Liuwei Dihuang, an anti-oxidant TCM formula comprised of six different herbs, increased maximal lifespan significantly with a dose of 4 ppm, but showed no significant effect at a higher dose of 7 ppm (Chen et al., 2019). Healthspan parameters, including frailty and cognitive function, were improved by extracts of the medicinal mushroom *Hericium erinaceus*, when given for 2 months to 21–23 months old male mice (Roda et al., 2021). Their healthspan measure was an alternative version of a frailty index, the locomotor frailty index which is determined via average speed and resting time. Their study found a 10% reduction in frailty in the treatment group and no sex-specific effects were observed. In a similar study design, the anti-oxidant acer truncatum seed oil was shown to improve cognitive function in male mice as measured with the Morris Water Maze test (Li et al., 2021). These results are promising and show the potential of medicinal herbs and their bioactive components as geroprotectors. However, further research is needed to understand what exactly is mediating the beneficial effects, especially in formulas such as Liuwei Dihuang, which mixes six different herbs.

**TABLE 5:** Study details and results for interventions with traditional Chinese medicine.

Study	Compound and dose	Start-age and duration	Strain	Outcome lifespan		Outcome healthspan				
				Median lifespan	Max lifespan	Frailty	Muscle function and coordination	Cognitive function and learning	Metabolism	Cancer
Roda et al. (2021)	*Hericium erinaceus*, 1 mg/day (orally)	21–23 months, for 2 months	C57BL/6 J	n.m	n.m	↑ m	n.m	↑ m	n.m	n.m
Li et al. (2021)	Acer truncatum seed oil, 0.01 mL/g/day (orally)	20 months, for 1 month	C57BL/6	n.m	n.m	n.m	n.m	↑ m	n.m	n.m
Chen et al. (2019)	Liuwei Dihuangh, 0.432 g/kg/day (water)	22–23 months, until end of life	C57BL/6 J	n.m	↑ (?) (*p* = 0.048)	n.m	n.m	n.m	n.m	n.m
	Liuwei Dihuangh, 0.72 g/kg/day (water)			n.m	= (?) (*p* = 0.078)					
S.-Q. Zhang et al., 2015)	Icariin, diet with 0.02%	12 months, until end of life	C57BL/6	↑ 8% m (*p* = 0.03)	= m	n.m	n.m	n.m	n.m	n.m
		12 months, for 12 months		n.m	n.m	n.m	↑ m	n.m	n.m	n.m

Notes: f, female; m, male; n.m., outcome was not measured (?), the sex was not specified; The arrows denote a significant improvesment (↑) or worsening (↓) of the respective outcome in the intervention group relative to the control group, while a (=) denotes no difference to control.

### Vitamins, supplements, antioxidants, and other compounds

Many vitamins, supplements, and antioxidants have been studied for their ability to improve healthspan and/or lifespan, as shown in Table 6. Interestingly, the strains of mice used in these studies vary beyond just the standard C57BL/6 mice commonly used in biomedical and aging studies (Palliyaguru et al., 2021b). In the case of SkQ1, for example, the authors used three different strains of mice (Anisimov et al., 2011) which ensures any effects observed are tested across different genetic backgrounds. A number of these compounds, which were tested in UM-HET3 mice as part of the Interventions Testing Program, did not have any significant effects on lifespan outcomes in either sex (Miller and Chrisp, 1999; Strong et al., 2013), with the exceptions of methylene blue and nordihydroguaiaretic acid (NHGA) showing sexually dimorphic lifespan effects (Strong et al., 2008; Harrison et al., 2014). Methylene blue improved lifespan only in female mice (Harrison et al., 2014), while NHGA improved only male lifespan (Strong et al., 2008; Harrison et al., 2014). Healthspan metrics were not measured in these animals. Additionally, glycine, which was also tested as part of the Interventions Testing Program, increased median lifespan by 4% in females and by 6% in males and significantly reduced the risk for lung adenocarcinomas (Miller et al., 2019).

**TABLE 6:** Study details and results for interventions with vitamins, supplements, antioxidants, and other compounds.

Study	Compound and dose	Start-age and duration	Strain	Outcome lifespan		Outcome healthspan				
				Median lifespan	Max lifespan	Frailty	Muscle function and coordination	Cognitive function and learning	Metabolism	Cancer
Xu et al. (2021)	PCC1, 20 mg/kg biweekly (orally)	20 months, for 4 months	C57BL/6 J	n.m	n.m	n.m	↑ m	n.m	n.m	n.m
		24–27 months, until end of life		↑ 64.2% m (*p* < 0.0001)	↑ 9.4% m (*p* < 0.0001)	n.m	= m	n.m	n.m	= m
Wirth et al. (2021)	Spermidine, 3 mM ad libitum (water)	17 months, for 6 months	C57BL/6 J	n.m	n.m	↑ m (only hair loss)	n.m	n.m	= m	n.m
			Rj							
Grohn et al. (2021)	C_60_ in olive oil, 1.7 mg/kg (injection), for 1 week daily, then for 1 month weekly, then for 7 months biweekly	25–27 months, for 7 months	CB6F1	= f	= f	n.m	n.m	n.m	n.m	n.m
	C_60_ in extra virgin olive oil, 4 mg/kg*bw/day (orally), for 1 week daily, then for 1 month weekly, then for 7 months biweekly	23 months, for 8 months	C57BL/6	= f and m	= f and m	n.m	= f and m	n.m	n.m	n.m
Li et al. (2022)	Sodium rutin, 0.2 mg/mL *ad libitum* (water)	8 months, until end of life	C57BL/6	↑ m (*p* < 0.01)	= m (trend, 3 months longer)	↑ m (kyphosis, cataract, hair loss)	↑ m	↑ m	n.m	n.m
Harrison et al. (2021)	Nicotinamide riboside, 1,000 ppm	8 months, until end of life	UM-HET3	= f and m	= f and m	n.m	n.m	n.m	n.m	n.m
Shahmirzadi et al. (2020)	Alpha-ketoglutarate, 2% w/w (food)	18 months, until end of life	C57BL/6 J	↑ 10.5%/16.6% f	↑ 19.7%/8% f	↑ f and m	↑ f and m (gait and activity, not treadmill)	n.m	n.m	n.m
				↑ 9.6%/12.8% m (cohort 1/2)	= m (cohort 1/2)					
Berry et al. (2020)	Trehalose, 0.1 mg/day (water)	25 months, for 1 month	C57BL/6N	n.m	n.m	n.m	= /↑ m (only coordination, not strength)	n.m	n.m	n.m
Miller et al. (2019)	Glycine, 8% in food	9 months, until end of life	UM-HET3	↑ 4% f (*p* = 0.006)	= f	n.m	n.m	n.m	n.m	↑ f and m
				↑ 6% m (*p* = 0.002)	↑ 6% m (*p* = 0.0005)					
Mitchell et al. (2018)	Nicotinamide, 37.5 mg/g*bw/day (food)	12 months, until end of life	C57BL/6 J	= m	= m	n.m	= m	= m	↑ m (only glucose)	n.m
	Nicotinamide, 75 mg/g*bw/day (food)			= m	= m	n.m	= m	= m	= m	n.m
Zhang et al. (2016)	Nicotinamide riboside, 400 mg/kg/day (food)	24 months, until end of life	C57BL/6JRj	n.m	↑ (?) (*p* = 0.034)	n.m	n.m	n.m	n.m	n.m
		22–24 months, for 6 weeks	C57BL/6 J	n.m	n.m	n.m	↑ (?)	n.m	n.m	n.m
Asseburg et al. (2016)	Polyphenol-rich grape skin extract, 200 mg/kg*bw/day (ST: orally, LT: food, LS: water)	22–24 months, for 6 weeks	C57BL/6 J	n.m	n.m	n.m	↑ (?)	n.m	n.m	n.m
		13 months, for 6 months (LT)		n.m	n.m	n.m	= m	n.m	n.m	n.m
		6 months, until end of life (LS)		= m	= m	n.m	↑ m (only locomotor activity)	n.m	n.m	n.m
Weimer et al. (2014)	D-Glucosamine	25 months, until end of life	C57BL/6NRj	n.m	↑ m and f (*p* = 0.0143)	n.m	n.m	n.m	↑ m and f	n.m
Harrison et al. (2014)	Nordi-hydroguaiaretic acid, 800 ppm (food)	6 months, until end of life	UM-HET3	↑ m (*p* = 0.04)	n.m	n.m	n.m	n.m	n.m	n.m
	Nordi-hydroguaiaretic acid, 2500 ppm (food)			↑ m (*p* = 0.0053)	n.m	n.m	n.m	n.m	n.m	n.m
	Nordi-hydroguaiaretic acid, 5,000 ppm (food)			= f	n.m	n.m	n.m	n.m	n.m	n.m
				↑ m (*p* = 0.0048)						
	Methylene blue, 28 ppm (food)	4 months, until end of life		= f (*p* = 0.17)	↑ f (*p* = 0.004)	n.m	n.m	n.m	n.m	n.m
				= m (*p* = 0.27)	= m (*p* = 0.6)					
Strong et al. (2013)	Resveratrol, 300 ppm (food)/50 mg/kg*bw/day	4 months, until end of life	UM-HET3	= f and m	= f and m	n.m	n.m	n.m	n.m	n.m
	Green tea extract, 2000 ppm (food)/333 mg/kg*bw/day			= f and m	= f and m	= f and m	n.m	n.m	n.m	n.m
	Curcumin, 2000 ppm (food)/333 mg/kg*bw/day			= f and m	= f and m	n.m	n.m	n.m	n.m	n.m
	Oxaloacetic acid, 2200 ppm (food)/367 mg/kg*bw/day			= f and m	= f and m	n.m	n.m	n.m	n.m	n.m
	Medium-chain triglyceride oil, 60′000 ppm (food)/10′000 mg/kg*bw/day			= f and m	= f and m	n.m	n.m	n.m	n.m	n.m
R. A. Miller et al., 2011)	Resveratrol, 300 or 1,200 ppm (food)	12 months, until end of life	UM-HET3	= f andm	= f andm	n.m	= f andm	n.m	n.m	n.m
Anisimov et al. (2011)	SkQ1, 5 or 250 nmol/kg/day water?	lifelong	129/sv	= f	n.m	n.m	n.m	n.m	n.m	n.m
	SkQ1, 1 or 30 nmol/kg/day water? (analyzed together)		BALB/c	= f	n.m	n.m	n.m	n.m	n.m	n.m
				↑ m (*p* < 0.05)						
	SkQ1, unknown dose		C57BL/6	= f	n.m	n.m	n.m	n.m	n.m	n.m
				↑ m (*p* < 0.05)						
Soda et al. (2009)	Polyamine high (Spermidine 1,540 nmol/g, Spermine 374 nmol/g)	3 months, for 19 months	Jc1:ICR	↑ m (*p* = 0.011, compared to normal and low)	n.m	n.m	n.m	n.m	n.m	n.m
	Polyamine normal (Spermidine 434 nmol/g, Spermine 160 nmol/g)			= m (*p* = 0.432, normal vs low)	n.m	n.m	n.m	n.m	n.m	n.m
	Polyamine low (Spermidine 224 nmol/g, Spermine 143 nmol/g)			= m (*p* = 0.432, normal vs low)	n.m	n.m	n.m	n.m	n.m	n.m
Strong et al. (2008)	Nordihydro-guaiaretic acid, 2500 ppm (food)	9 months, until end of life	UM-HET3	= f	= f and m	n.m	n.m	n.m	n.m	n.m
				↑ m (*p* = 0.0006)						
Pearson et al. (2008)	Resveratrol, 100 ppm (food)	12 months, until end of life	C57BL/6NIA	= m	= m	= m	= m	n.m	n.m	n.m
	Resveratrol, 400 ppm (food)			= m	= m	↑ m (less cataracts)	↑ m (improved rotarod)	n.m	n.m	n.m
	Resveratrol, 2400 ppm (food)			= m	= m	n.m	n.m	n.m	↑ m (lower cholesterol)	n.m
Kitani et al. (2007)	Tetrahydro-curcumin, 0.2% (food)	13 months, until end of life	C57BL/6JHsd	↑ m (*p* < 0.01)	↑ m (*p* < 0.01)	n.m	n.m	n.m	n.m	n.m
		19 months, until end of life		= m	= m					
	Green tea polyphenols, 80 mg/L (water)	13 months, until end of life		↑ m (*p* < 0.05)	= m					
Navarro et al. (2005)	Vitamin E (dl-RRR-α-tocopherol, 5 g/kg (food)	7 months, until end of life	CD-1	= f	= f	n.m	↑ m	↑ m	n.m	n.m
				↑ m (*p* < 0.0001)	↑ m (*p* < 0.0001)					
Lee et al. (2004)	α-lipoic acid, 600 ppm (food)	14 months, until end of life	B6C3F_1_	= m	= m	n.m	n.m	n.m	n.m	= m
	Coenzyme Q_10_, 100 ppm (food)			= m	= m	n.m	n.m	n.m	n.m	= m
Anisimov et al. (2003)	Melatonin, 2 mg/L (5x per months, water)	3 months, until end of life	SHR	= f	= f	n.m	n.m	n.m	n.m	↑ f
	Melatonin, 20 mg/L			= f	↑ f (*p* < 0.05, last 10%)	n.m	n.m	n.m	n.m	= f
Morley and Trainor (2001)	Vitamin E, 20, 40 and 400 mg/kg (food)	Conception, until end of life	Balb/c	= f	= f	n.m	n.m	n.m	n.m	n.m
Khavinson et al. (2000)	Vilon (Lys-Glu), 0.1 mg (5x per months, injection)	6 months, until end of life	CBA	= f	↑ f (*p* < 0.05, last 10%)	n.m	↑ f	n.m	n.m	↑ f
Khavinson and Anisimov (2000)	Vilon (Lys-Glu), 0.1 mg (5x per months, injection)	6 months, until end of life	CBA	= f	↑ f (*p* < 0.05, last 10%)	n.m	↑ f	n.m	n.m	↑ f
	Epithalon (Ala-Glu-Asp-Gly), 0.1 mg (5x per months, injection)			↑ f (*p* < 0.05)	= f	n.m	↓ f	n.m	n.m	↑ f
Miller and Chrisp (1999)	DHEA sulfate, 100 mg/mL (water)	Birth, until end of life	UM-HET3	= f and m	= f and m	n.m	n.m	n.m	n.m	= f and m
Saito et al. (1998)	N-tert-butyl-a-phenylnitrone, 0.25 mg/mL (water)	24.5 months, until end of life	C57BL/6 J	↑ m (*p* < 0.005, mean ls)	n.m	n.m	n.m	n.m	n.m	n.m
Lönnrot et al. (1998)	Ubiquinone Q10, 10 mg/kg/day	2 months, until end of life	C57/B17	= m	= m	n.m	n.m	n.m	n.m	n.m
Bezlepkin et al. (1996)	Antioxidant mixture: 7.5 mg beta carotene, 15 mg *α*-tocopherol, 50 mg ascorbic acid, 25 mg rutin, 25 μg selenium, 5 mg zinc per kg*bw	2 months, until end of life	C57BL/6	↑ m (*p* < 0.05, mean ls)	↑ m (*p* < 0.05, last 10%)	n.m	n.m	n.m	n.m	n.m
		9 months, until end of life		↑ m (*p* < 0.05, mean ls)	↑ m (*p* < 0.05)					
		16 months, until end of life		= m	= m					
		23 months, until end of life		= m	= m					
Heidrick et al. (1984)	2-mercaptoethanol, 0.25% of food	4 months, until end of life	BC3F_1_	↑ m 13.2% (*p* < 0.005, mean ls)	↑ m (*p* < 0.001, last 10%)	n.m	n.m	n.m	n.m	↑ m
Davies and Schofield (1980)	β-aminopropio-nitrile, 0.5–2 mg/mL water	3–4 months, until end of life	C57BL/Icrfa	↓ f	↓ f	n.m	n.m	n.m	n.m	n.m
	β-aminopropio-nitrile, 1 mg/mL water	9 months, until end of life		= f	= f					
Miquel and Economos (1979)	Magnesium thiazolidine carboxylate, 0.07% of food	23 months, until end of life	C57BL/6	↑ f 7% (no *p*-value)	n.m	n.m	n.m	n.m	n.m	n.m
LaBella and Vivian (1978)	β-aminopropio-nitrile, 1 or 3 mg/mL water	2 months, for 6/12/18 months	LAF/J	↑ m (*p* < 0.05, mean ls)	= m	n.m	n.m	n.m	n.m	n.m
	β-aminopropio-nitrile, 3 mg/mL water	2 months, for 6 months		= m	= m					
Comfort et al. (1971)	Ethoxyquin, 0.5% of food	3 months, until end of life	C3H	↑ f and m (*p* < 0.005, not specified)	n.m	n.m	↑ f and m	n.m	n.m	= f and m

Notes: f, female; m, male; n.m., outcome was not measured (?), the sex was not specified; The arrows denote a significant improvement (↑) or worsening (↓) of the respective outcome in the intervention group relative to the control group, while a (=) denotes no difference to control. PCC1, procyanidin C1; DHEA, dehydroepiandrosterone.

Of the studies that used standard C57BL/6 mice, several found improvements in lifespan as well as healthspan metrics. Alpha-ketoglutarate was tested in both female and male mice and showed positive effects on lifespan and healthspan in a sex-independent manner, although stronger lifespan effects were observed in female mice (Shahmirzadi et al., 2020). Healthspan showed similar effects in both sexes, with particularly good improvements in female fur color. A sex-independent increase in median and maximal lifespan was also achieved with D-glucosamine, with a treatment onset at 25 months, and additional improvements in glucose metabolism were observed (Weimer et al., 2014). Treatment with procyanidin C1 (PCC1) from grape seeds (Xu et al., 2021), as well as treatment with sodium rutin, a flavonoid (Li et al., 2022), in male mice increased lifespan and several measures of healthspan, such as frailty, muscle function, and cognitive function. Multiple other studies measuring solely lifespan in C57BL/6 mice showed improvements with compounds including antioxidants and polyphenol mixtures, however, most were tested in male mice only (Bezlepkin et al., 1996; Saito et al., 1998; Kitani et al., 2007). Future studies with these compounds should involve healthspan outcomes as well as validating findings in female mice.

Declining NAD levels with age are thought to be one contributor to age-related degeneration (Gomes et al., 2013; McReynolds et al., 2020), with supplementation of NAD precursors evaluated as therapeutic avenues. In C57BL/6 mice, 400 ppm of nicotinamide riboside increased the maximal lifespan as well as muscle function and coordination in mice when given late in life (22–24 months) (Zhang et al., 2016), however, sex of the mice in this study was not specified. A higher dose of 1,000 ppm of nicotinamide riboside started early in life (8 months) did not improve lifespan parameters in female and male UM-HET3 mice (Harrison et al., 2021). Supplementation with nicotinamide started at 12 months improved glucose metabolism but was also not able to improve lifespan parameters in male C57BL/6 mice (Mitchell et al., 2018).

The already mentioned SkQ1, which was tested in different genetic backgrounds, showed sexually dimorphic effects in C57BL/6 mice as well as BALB/c mice, where it was only able to extend male, but not female lifespan (Anisimov et al., 2011). Of the compounds in this category, magnesium thiazolidine carboxylate was the only compound that extended female median lifespan, even though *p*-values are missing in this study (Miquel and Economos, 1979). Studies testing spermidine, trehalose, polyphenol-rich grape skin extract, and resveratrol found improvements in several healthspan metrics in male C57BL/6 mice (Pearson et al., 2008; Asseburg et al., 2016; Berry et al., 2020; Wirth et al., 2021), however, they either observed no effects on lifespan (Pearson et al., 2008; Asseburg et al., 2016; Wirth et al., 2021), or lifespan was not measured (Berry et al., 2020). Of note, a number of these studies included males only. No effects on lifespan or healthspan could be shown with C_60_ in olive oil in either female or male C57BL/6 mice (Grohn et al., 2021). Interestingly, b-aminopropionitrile was shown to reduce female lifespan (Davies and Schofield, 1980), despite an earlier study reporting it increased lifespan in male LAF/J mice (LaBella and Vivian, 1978). This highlights the importance of using both sexes and a variety of mouse strains. Studies on vitamin E discovered an interesting sexual dimorphism, as it only had an effect on male, but not female lifespan (Morley and Trainor, 2001; Navarro et al., 2005). Vilon and Epithalon, both synthetic peptides, were shown to significantly improve female lifespan as well as multiple metrics of healthspan (Khavinson and Anisimov, 2000; Khavinson et al., 2000). Anisimov et al. also observed an interesting effect of melatonin on uncoupling lifespan and healthspan. At a low dose (2 mg/L), tumor incidence was reduced, but lifespan was unaffected; meanwhile at a higher dose (20 mg/L), lifespan was increased, but tumor incidence was unaffected (Anisimov et al., 2003). Dose-dependent effects were also observed by Soda and colleagues (Soda et al., 2009) when testing a combination of polyamines (spermidine and spermine), with the highest dose improving lifespan when compared to lower doses, which were solely tested in male mice. Further, 2-mercapto-ethanol was shown to improve lifespan and healthspan, but it was only tested in male mice (Heidrick et al., 1984). Ethoxyquin, a quinoline-based antioxidant, was shown to improve lifespan and healthspan in both males and females (Comfort et al., 1971). However, interventions with ubiquinone (Lönnrot et al., 1998; Lee et al., 2004) and alpha-lipoic acid in male mice did not show any effects on lifespan (Lee et al., 2004). While some of the discussed compounds have shown promising results in extending lifespan and improving healthspan in mice, most studies have only been conducted on one sex, leaving questions about their potential benefit on the opposite sex.

### Adherence to the SABV mandate for preclinical studies

**FIGURE 3:**
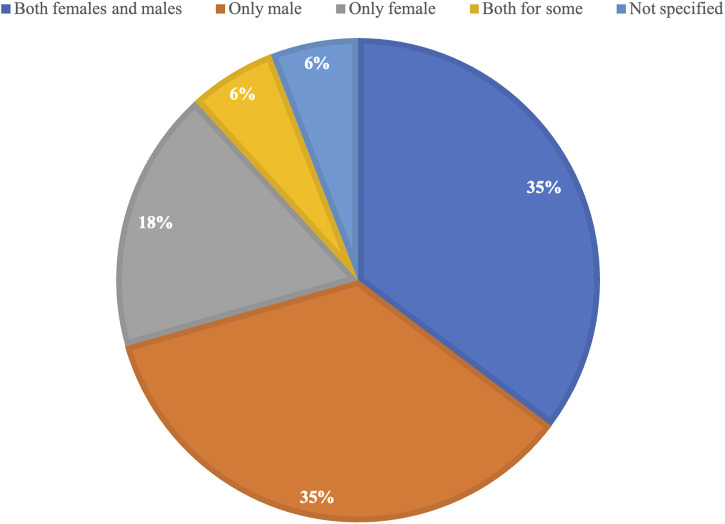
Proportional representation of the use of female and male mice in all studies included in this manuscript since 2020.

Since 2020, the NIH mandate has been in place, which requires authors to include both males and females in NIH-funded preclinical research studies. Of the 17 studies published since 2020 included in this systematic review, only six used both female and male mice and compared their outcomes (35%) (Figure 3). This is the same number of studies that used only male mice for their research (35%). When looking at the proportion of studies that used both sexes in all included studies, which is 36% (26/72) (Figure 2), nothing has changed despite the mandate being in place. This highlights the need for journals to further encourage or require compliance with the mandate and to promote the integration of sex as a biological variable in preclinical research studies. This could lead to a better understanding of the potential sex-specific differences in the outcomes of these studies and lead to improved treatments for all patients, regardless of their biological sex. It is also important for researchers to be aware of the potential impact of sex on the outcomes of their studies and to design studies that accurately represent the populations they aim to serve.

## Discussion

The goal of this manuscript was to systematically review the available literature on sexual dimorphism in the use of pharmacological compounds as potential geroprotectors. We focused on lifespan and healthspan outcomes using mice as a model organism. Of the more than 7000 potentially eligible studies identified through our search, only 72 original research publications met the stringent inclusion and exclusion criteria.

Our results showed that of the 72 included studies, 40% (29/72) of studies only used male mice or did not clarify the sex, 20% (14/72) of studies used only female mice, and only 36% (26/72) of studies used both sexes for all their measurements (Figure 2). Additionally, of all studies using both sexes, 73% (19/26) showed sex-specific outcomes. These data highlight the importance of considering sex as a biological variable (SABV) when testing novel geroprotector interventions. The failure to do so prevents a clear understanding of the sex-specific effects of the tested compounds, particularly as our systematic review found that 73% of studies showed sex differences in the effects of the tested compound on the health or lifespan outcome. It is tempting to speculate how many geroprotectors that have “failed” preclinical testing may have been successful if they were tested in females.

In 2016, the National Institutes of Health mandate came into force, requiring the use of both males and females in NIH-funded research, unless there was a strong scientific justification. This mandate resulted from the workshop on sex as a biological variable. Since then, a number of authors (Garcia-Sifuentes and Maney, 2021; Shansky and Murphy, 2021; Carmody et al., 2022) have looked at adherence to these policies across different scientific disciplines, with a general consensus that adherence should be improved. In addition to inclusion, it is important that authors also provide statistical evidence supporting the difference. A recent report examining sex differences across nine biological disciplines (in 147 articles) found incorrect use of statistics by authors to support their claims, which they suggest may lead to over-reporting or masking of sex-specific differences (Garcia-Sifuentes and Maney, 2021). These examples argue for continuing discussion on the importance of SABV and ongoing efforts to train biomedical researchers in how to test for and report sex differences correctly in their studies. It may be of importance for leading SABV journals to put together a white paper detailing the best practice for incorporating SABV in biomedical research, including how to appropriately use statistical tests to report effects, much like the PRISMA guidelines for systematic reviews. It is, however, encouraging to see journals such as the American Journal of Physiology-Heart and Circulatory Physiology requiring the inclusion of sex as a biological variable in the reporting of published articles (Denfeld et al., 2022) in their journal. Other journals, such as the Journals of Gerontology and Arteriosclerosis, Thrombosis, and Vascular Biology, have published statements recommending this to their authors (Le Couteur et al., 2018; Robinet et al., 2018). In the studies included here and published since 2020, there is no change observable regarding the use of both sexes when compared to all studies that were included.

Two more recent studies and therefore not yet included in this review have implemented the use of both sexes and found improvements in lifespan as well as healthspan. In the first study, the NADase CD38 inhibitor 78c increased median lifespan by 17% in males, but not in females, and improved exercise performance, endurance, and metabolic function in males (Peclat et al., 2022). In the second study, the PI3K p110α inhibitor, which targets the insulin receptor/insulin-like growth factor receptor pathway, extended median and maximal lifespan of both male and female mice and improved muscle function, with more significant effects in females (Hedges et al., 2023). These results further emphasize the importance of considering biological sex in preclinical research.

While including both sexes in preclinical research is critical, it is equally important to consider the genetic diversity of the mouse strains used in these studies. Testing interventions in heterogenous mouse strains provides a more accurate representation of how treatments may perform in a diverse human population, improving our ability to develop safe and effective treatments. Studies comparing genetically diverse inbred mouse strains have found significant differences in lifespan parameters (Yuan et al., 2009; Yuan et al., 2020), highlighting the importance of using multiple mouse strains when researching a potential geroprotector. While the studies included in this review exhibit some level of genetic diversity, there is room for improvement in terms of testing a specific compound on several genetic backgrounds to ensure greater generalizability.

In addition to the healthspan parameters focused on in this review, there are further health assessments that can be useful in intervention studies in aging mice. These include blood chemistry analysis, which provides information on glucose homeostasis, lipid metabolism, liver and kidney function, and inflammatory markers (O’Connell et al., 2015; Palliyaguru et al., 2021a; Zhang et al., 2022). Live animal imaging techniques, such as magnetic resonance imaging (MRI) (Chen et al., 2011) and positron emission tomography (PET) (Borrás et al., 2011; Hulsmans et al., 2018), can allow for the non-invasive visualization of organs and tissue and can therefore provide insights into structural and functional changes occurring with an intervention. Analysis of metabolomics (Adav and Wang, 2021; Tian et al., 2022), proteomics and transcriptomics (Takemon et al., 2021) can be used to identify changes in metabolic pathways, protein expression and gene expression in response to an intervention. Finally, tissue histology can assess changes on a tissue and cellular level (Pettan-Brewer and Treuting, 2011). Generally, it is important to use a wide variety of health assessment tools to get a more comprehensive understanding of the efficacy of geroprotective interventions.

### Limitations of the systematic review

There are a number of limitations to consider when interpreting the findings of this systematic review. One limitation is that only one database (Pubmed) was used, which means that there may be a selection bias, as the studies included in the review may not be representative of the overall population of geroprotector studies. Additionally, the studies included in the review used a variety of outcomes and statistical methods, making it harder to compare the results across studies. Some studies also had missing information, which can impact the ability to accurately interpret the results. Furthermore, the quality of the studies included in the review may vary, with some studies having more robust designs, higher statistical power, and more reliable results compared to others. Overall, these limitations should be considered when interpreting the results of the review and planning future research on geroprotectors and their effects on healthspan and lifespan.

## Conclusion

Pharmacological interventions represent an attractive therapeutic avenue for modulating age-related diseases and frailty, especially in those individuals for whom dietary interventions are not feasible. The results from our systematic review show that most studies have only been performed in males, meaning the generalizability of these findings to females is unknown. Given that females represent roughly 50% of the population, the knowledge gap surrounding the translational value of these interventions is large, as for half the population we do not know how these may impact healthspan or lifespan. Thus, we reiterate the point that only by studying both males and females can we leverage sex-specific differences to provide novel insights into the pathophysiology of aging and improve healthy aging for all.

## Data Availability

The original contributions presented in the study are included in the article/Supplementary Material, further inquiries can be directed to the corresponding authors.
